# Improving Serodiagnosis of Human and Canine Leishmaniasis with Recombinant *Leishmania braziliensis* Cathepsin L-like Protein and a Synthetic Peptide Containing Its Linear B-cell Epitope

**DOI:** 10.1371/journal.pntd.0003426

**Published:** 2015-01-08

**Authors:** Daniel Menezes-Souza, Tiago Antônio de Oliveira Mendes, Matheus de Souza Gomes, Daniella Castanheira Bartholomeu, Ricardo Toshio Fujiwara

**Affiliations:** 1 Departamento de Parasitologia, Instituto de Ciências Biológicas, Universidade Federal de Minas Gerais, Belo Horizonte, Brazil; 2 Instituto de Genética e Bioquímica, Universidade Federal de Uberlândia, Patos de Minas, Brazil; University of Pittsburgh, United States of America

## Abstract

**Background:**

The early and correct diagnosis of human leishmaniasis is essential for disease treatment. Another important step in the control of visceral leishmaniasis is the identification of infected dogs, which are the main domestic reservoir of *L. infantum.* Recombinant proteins and synthetic peptides based on *Leishmania* genes have emerged as valuable targets for serodiagnosis due to their increased sensitivity, specificity and potential for standardization. Cathepsin L-like genes are surface antigens that are secreted by amastigotes and have little similarity to host proteins, factors that enable this protein as a good target for serodiagnosis of the leishmaniasis.

**Methodology/Principal Findings:**

We mapped a linear B-cell epitope within the Cathepsin L-like protein from *L. braziliensis*. A synthetic peptide containing the epitope and the recombinant protein was evaluated for serodiagnosis of human tegumentary and visceral leishmaniasis, as well as canine visceral leishmaniasis.

**Conclusions/Significance:**

The recombinant protein performed best for human tegumentary and canine visceral leishmaniasis, with 96.30% and 89.33% accuracy, respectively. The synthetic peptide was the best to discriminate human visceral leishmaniasis, with 97.14% specificity, 94.55% sensitivity and 96.00% accuracy. Comparison with *T. cruzi*-infected humans and dogs suggests that the identified epitope is specific to *Leishmania* parasites, which minimizes the likelihood of cross-reactions.

## Author Summary

Leishmaniasis is one of the major diseases of importance in public health and its precise diagnosis may represent one of the most relevant challenges for the control and possible eradication of the disease. In this context, recombinant proteins and synthetic peptides based on *Leishmania* genes have emerged as valuable targets for serodiagnosis due to their increased sensitivity, specificity and potential for standardization. Cathepsin L-like (CatL) genes are more abundant in stationary promastigotes and amastigotes, and have less than 40% identity with human proteins and more than 60% identity with other *Leishmania* species. We mapped a linear B-cell epitope in the CatL protein sequence and compared its performance with the recombinant protein and current serology methodologies for the diagnosis of human tegumentary and visceral leishmaniasis as well as of canine visceral leishmaniasis (CVL). Both the recombinant protein and synthetic peptide showed higher specificity and sensitivity than crude preparations commonly used for other antigens, and thus, they are valuable targets to compose an antigen panel that could significantly improve leishmaniasis diagnosis.

## Introduction

Leishmaniasis is a complex disease with cutaneous, mucocutaneous and visceral forms, and it is caused by protozoan parasites of the genus *Leishmania*. The disease is present in more than 98 countries, with approximately 0.2 to 0.4 million and 0.7 to 1.2 million cases of visceral (VL) and tegumentary (TL) leishmaniasis, respectively, occurring each year [1], [2]. Currently, the disease is expanding to non-endemic areas such as Canada, the United States, Italy and Germany [3], [4]. The early and correct diagnosis of VL is important for reducing mortality, as the disease is lethal if left untreated. Another important step in the control of VL is the identification of infected dogs, which are the main domestic reservoir of *L. infantum* [5]. Although the tegumentary diseases are often non-lethal, there are grave consequences for the patient [6] that can be prevented with rapid and accurate diagnosis and treatment.

Currently, the diagnosis of TL is based on Montenegro skin test [7], [8] that identifies a delayed-type hypersensitivity response to parasite antigens in infected individuals and directly detects parasites in lesions [9], [10]. Canine and human visceral leishmaniasis diagnosis requires the identification of clinical symptoms and serological tests. Crude *Leishmania* antigen preparations including soluble antigens are the most common parasite proteins employed in immunodiagnosis of leishmaniasis. Serological techniques performed with this antigen have high sensitivity, but they lack specificity [11]. False positive results are frequently observed in sera from humans and dogs infected with *T. cruzi* [12]–[14]. Additionally, the different parasite strains and protocols used for crude antigen preparations cause variations that may affect the sensitivity of this method.

Improving serological tests for Leishmaniasis diagnosis is important because they are rapid, easy to perform and can easily be implemented under the conditions commonly encountered in developing countries [15], [16]. Furthermore, serological tests can potentially diagnose infections before lesions are formed by tegumentary disease [17]. Recombinant *Leishmania* proteins have emerged as valuable targets for serodiagnosis due to their increased sensitivity, specificity and potential for standardization [17]. In this context, various recombinant proteins, among them k39, KMPII, Peroxidoxins, LACK, nucleosomal histones (H2A, H2B, H3 and H4) and heat shock proteins (families 60, 70 and 83) have been tested in the diagnosis of visceral and tegumentary leishmaniasis and obtained promising results for development of diagnosis kits using these antigens [17]–[24]. Moreover, *in silico* and experimental methods for epitope mapping and peptide synthesis have great potential for the discovery of new potential pathogen antigens [25], [26]. Chemically synthesized peptides have low costs and high specificity and are also free of contaminants from bacteria or other host cells that are frequently used to produce recombinant proteins [27], [28].

Cysteine proteases have been implicated in several processes during parasite life cycles, including interaction with host cells and immune evasion. In *Leishmania* parasites, Cathepsin L-like (CatL) genes are more abundant in stationary promastigotes and amastigotes [29], and the mature protein is both surface-associated [30] and secreted [31]. Knockout studies of this protein in *L. mexicana* and *L. infantum* demonstrate its importance for parasite survival inside macrophages; for example, it modulates host immune responses [32]–[34]. Beyond expression in the intracellular stage, CatL proteins have less than 40% identity with human proteins and more than 60% identity with other *Leishmania* species. Hence, this protein is a good target for serodiagnosis.

We evaluated the potential use of *L. braziliensis* CatL protein for the serodiagnosis of human tegumentary and visceral leishmaniasis as well as of canine visceral leishmaniasis (CVL). Furthermore, we mapped a linear B-cell epitope in the CatL protein sequence and compared its performance with the recombinant protein using current serology methodologies. Finally, we evaluated the reactivity of the CatL epitope with human and canine sera.

## Methods

### Ethics statement and sera samples

Experiments involving dog samples were performed in compliance with the guidelines of COBEA (Brazilian College of Animal Experimentation), strictly followed the Brazilian law for “Procedures for the Scientific Use of Animals” (11.794/2008) and were approved by the Institutional Animal Care and Committee on Ethics of Animal Experimentation (Comitê de Ética em Experimentação Animal – CETEA) from the Federal University of Minas Gerais (protocol number 44/2012). The use of human samples was approved by the Ethics Committee of the Federal University of Minas Gerais (protocol CAAE – 00842112.2.0000.5149). All subjects provided written informed consent before blood collection.

A total of 65 sera samples were obtained from TL patients from the Centro de Referência em Leishmaniose (Januária, Minas Gerais, Brazil), of which 45 and 20 patients presented cutaneous (CL) and mucosal (ML) clinical disease, respectively. Sera samples from 55 patients with VL were also obtained from the University Hospital (Montes Claros, Minas Gerais state, Brazil). Parasitological confirmation of *Leishmania* infection was performed by microscopic analysis of biopsies from cutaneous lesions (TL) or bone marrow aspirates (VL), and molecular detection of the parasite was performed by PCR using specific primers for *Leishmania* kDNA [35]. All *Leishmania*-infected patients are known to be uninfected with *T. cruzi*. Chagasic human sera was collected from 20 patients with *T. cruzi* infections, which were confirmed by hemoculture or the Chagatest recombinant ELISA v.3.0 kit in combination with the Chagatest hemagglutination inhibition (HAI) tests; the absence of *Leishmania* infection was also confirmed in these patients. Sera samples from 50 healthy humans from non-endemic *Leishmania* or *Trypanosoma* areas were used as negative controls.

Dog sera samples were obtained from the endemic area for CVL in Minas Gerais, Brazil. The infection was confirmed in 30 animals by the presence of amastigotes in bone marrow aspirates, as observed by microscopic analysis. Samples from 15 dogs experimentally infected with *T. cruzi* (CD) and negative for *Leishmania* were used to evaluate cross-reactivity. A total of 30 dogs from areas without endemic visceral leishmaniasis and negative for *Leishmania* and *T. cruzi* were included as a control group. To facilitate the visualization of various human and dog sera samples used in this study a flowchart was prepared and is shown in supplementary figure 1 (S1 Fig.).

**Figure 1:**
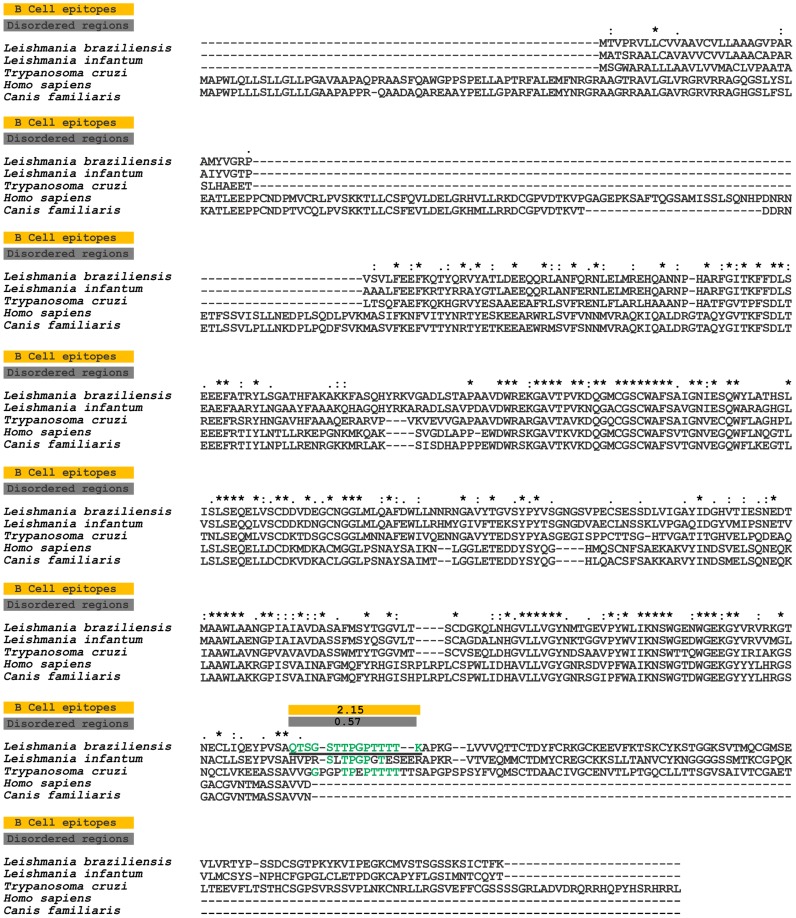
Sequence divergence and prediction of B-cell linear epitopes and intrinsically unstructured/disordered regions in *L. braziliensis* Cathepsin L-like and its orthologs. Alignment of *L. braziliensis* Cathepsin L-like (TritrypDB ID: LbrM.08.0830) and orthologous proteins in *L. infantum* (TritrypDB ID: LinJ.08.0960), *T. cruzi* (TritrypDB ID: TcCLB.509429.320), *H. sapiens* (RefSeq ID: NP_003784.2) and *C. familiaris* (RefSeq ID: XP_005631485.1). The yellow boxes mark predicted B-cell epitopes, and the gray boxes mark predicted disordered regions. The continuous black underlined amino acid sequences in the LbrM.08.0830 protein represent one potential B-cell epitopes predicted by Bepipred, and the colors highlight amino acids conserved between the *T. cruzi*, *C. familiaris* and *H. sapiens* sequences and *L. braziliensis*.

### Sequence analysis and linear B-cell epitope prediction

The sequences of *L. braziliensis* CatL protein (Gene ID: LbrM.08.0830) and its orthologs in *L. infantum* (Gene ID: LinJ.08.0960) and *T. cruzi* (Gene ID: TcCLB.509429.320) were obtained from TritrypDB [36]. The *Homo sapiens* (RefSeq ID: NP_003784.2) and *Canis familiaris* (XP_005631485.1) proteins most similar to the *L. braziliensis* sequence were identified using BLASTp [37] against the NCBI non-redundant protein database [38]. Multiple alignments of sequences were performed with ClustalX 2.0 [39] with the default parameters. To predict linear B-cell epitopes in the *L. braziliensis* CatL protein, the Bepipred program [40] was used with a cut-off of 1.3; at least 9 contiguous amino acids with individual prediction scores above the cutoff were considered as candidate epitopes. Intrinsically unstructured/disordered regions (IURs) were predicted using the IUPred program [41]. IURs were classified as at least 9 continuous amino acids with individual prediction scores above 0.5.

To analyze for epitope specificity, BLAST search, with parameters adjusted for short input sequence, using the epitope sequence as query was performed against all reported human and dog sequences from NCBI non-redundant protein database. The specific parameters were set to 20.000 expect threshold, the word size length was 2, PAM30 without compositional adjustment was used as substitution matrix and low complexity regions filter was switched off (parameters available at http://www.ncbi.nlm.nih.gov/blast/Why.shtml) [42]–[44].

### Soluble *Leishmania braziliensis* antigen (SLbA)

*L. braziliensis* strain MHOM/BR/75/M2904 promastigotes were grown to stationary phase at 24°C in Schneider's insect medium (Sigma-Aldrich) supplemented with 10% inactivated fetal bovine serum, 100 U/mL penicillin and 100 µg/mL streptomycin and pH adjusted to 7.2. A total of 1×10^10^ parasites were washed three times with cold phosphate buffered saline, followed by three cycles of freezing (liquid nitrogen) and thawing (42°C). After ultrasonication with 10 alternating cycles of 30 s at 35 MHz, the lysate was centrifuged at 6,000×*g* at 4°C for 15 min. The supernatant containing SLbA was collected, and the protein concentration was estimated using the Pierce BCA Protein Assay (Thermo Scientific).

### Cloning, recombinant protein expression and purification

To assess the potential use of *L. braziliensis* CatL protein in the diagnosis of human and canine leishmaniasis, we expressed this protein as a His-tagged recombinant protein. Initially, the CatL gene was PCR-amplified from *L. braziliensis* genomic DNA using forward (5′GCTAGCATGACGGTGCCGAGGGTC) and reverse (5′GGATCCCTACTTGAACGTGCAGATGCTCT) primers with *NheI* and *BamHI* restriction sites, respectively (underlined letters). The 1.33-kb fragment was excised from the gel, purified, digested with the restriction enzymes and ligated to a similarly digested *p*ET28a-TEV vector [45]. The recombinant plasmid was introduced to electrocompetent *E. coli* BL21 Arctic Express (DE3) cells (Agilent Technologies, USA) by electroporation using a MicroPulser Electroporation Apparatus (Bio-Rad Laboratories, USA). Gene insertion was confirmed by colony PCR and sequencing using T7 primers (Macrogen, South Korea). The recombinant CatL (*r*CATL) expression was performed by adding 1.0 mM IPTG (Isopropyl-β-D-thiogalactopyranoside, Promega, Canada) for 24 h at 12°C with shaking at 200 rev min^−1^. The cells were then lysed by sonication and centrifuged at 10,000×*g* for 30 min at 4°C. The recombinant CatL protein was purified using a HisTrap HP affinity column connected to an ÄKTAprime chromatography system (GE Healthcare, USA). The eluted fractions containing *r*CatL were concentrated using Amicon Ultra 15 Centrifugal Filters, 10,000 NMWL (Millipore, Germany), and further purified on a SuperdexTM 200 gel filtration column (GE Healthcare Life Sciences, USA).

### Peptide synthesis and purification

Soluble peptide was manually synthesized in the solid phase on a 30-µmol scale using 9-florenyl-methoxy-carbonyl (Fmoc) chemistry [46]. First, Fmoc-amino acids were activated with a 1∶2 solution of Oxyme and DIC. The activated amino acids were incorporated into a Rink amide resin with a substitution degree of 0.61. Fmoc deprotection was then performed using 25% 4-methylpiperidine. These steps were repeated until peptide synthesis was complete. The side-chain was deprotected and released from the resin by a solution of 9.4% trifluoroacetic acid, 2.4% water, and 0.1% triisopropylsilane. The peptide was precipitated with cold diisopropyl ether and purified by high-performance liquid chromatography (HPLC) on a C18 reverse-phase column using a gradient program of 0 to 25% acetonitrile. The peptides were obtained with 90% purity, as confirmed by mass spectrometry using Autoflex Speed MALDI/TOF equipment [25].

### ELISA and depletion ELISA

First, *r*CatL and SLbA were coated onto 96-well microplates (Nalge Nunc Intl., USA) overnight at 2–8°C at a concentration of 2.5 µg/mL for *r*CatL and 0.5 µg/mL for SLbA. For the peptide, flat-bottom plates (Costar, USA) were coated with 10 µg/well of soluble peptide overnight at 37°C. After blocking with BSA (0.05 g/mL) in PBS (pH adjusted to 7.2) for 1 hour at 37°C, the plates were washed three times with PBS containing Tween 20 (PBS-T; 0.5 µL/mL) and incubated with human or dog serum (1∶100 dilution). The plates were washed three times with PBS-T, and a secondary HRP-conjugated anti-human or anti-dog IgG antibody (1∶5,000) was added for 1 hour at 37°C, followed by four washes. The 3,3′,5,5′-Tetramethylbenzidine (TMB) substrate (Sigma-Aldrich, USA) in citrate buffer containing hydrogen peroxide was used for detection. The reaction was stopped after 30 min with 4 N H_2_SO_4_, and the absorbance was measured at 450 nm. For the depletion ELISA, the sera was incubated in peptide-coated and blocked plates at a 1∶100 dilution overnight at 2–8°C [23], [24]. Depleted and undepleted samples were transferred to plates coated overnight with *r*CatL (50 ng/well) and blocked. ELISAs were performed as described above. For ELISA assays, each serum sample was evaluated in duplicate.

### Statistical analysis

All of the statistical analyses were performed using GraphPad Prism release 5.0. The cut-off values for *r*CatL, SLbA and the synthetic peptide were established using the receiver-operator curve (ROC curve). The cut-off was chosen based on the point that provides the maximum sum of sensitivity and specificity [47]. The EIE-LVC cut-off was obtained according to the manufacturer's recommendation (twice the average of the negative control). Each test was evaluated for sensitivity (Se), specificity (Sp), positive predictive value (PPV), negative predictive value (NPV), area under curve (AUC) and accuracy (AC). The degree of agreement between the ELISA assays using *r*CatL, SLbA or the EIE-LVC Kit and the parasitological test (biopsy, aspirate or PCR) was determined by kappa index (κ) values with 95% confidence intervals and classified according to the Fleiss scale: 0.00–0.20, poor; 0.21–0.40, fair; 0.41–0.60, moderate; 0.61–0.80, good; 0.81–0.99, very good and 1.00, perfect. The normal distribution of data was evaluated by the Kolmogorov-Smirnov test. For depletion assays, significant differences were detected using a two-way ANOVA. The differences were considered to be statistically significant at *p*<0.05.

## Results

### Sequence divergence between host and parasite combined with epitope prediction suggest CatL protein as a diagnostic target

The most similar human and canine Cathepsin detected in the NCBI database had 28.34% and 28.12% identity, respectively, with *L. braziliensis* CatL (Table 1). This protein shares 65.99% and 47.60% identity and 70.34% and 56.63% of similarity with its orthologs in *L. infantum* and *T. cruzi*, respectively (Table 1). In relation to host orthologs, *L. braziliensis* CatL has 28.12% and 28.34% identity and 35.98% and 36.96% of similarity with *C. familiaris* and *H. sapiens*, respectively (Table 1). Only one B-cell linear epitope peptide was predicted by Bepipred program as B-cell linear epitope in the carboxy terminal region of *L. braziliensis* CatL protein (Fig. 1). This peptide is not present in the human and dog orthologs, but it has 40.00% and 53.30% of identity and 73.33% and 86.67% of similarity to *L. infantum* and *T. cruzi*, respectively. We also evaluated if other proteins from human and dog genomes could have a similar sequence to linear B-cell epitope of *L. braziliensis* CatL (S1–S2 Tables). No human or dog sequence is 100% similar to peptide-1. The most related human and dog sequences display 60% similarity with peptide-1 (2 for *H. sapiens* and 3 for *C. familiaris*) (S1–S2 Tables). This data suggest low probability of cross-reactivity with host proteins. The identified B-cell linear epitope is present in a predicted non-structured region; this characteristic is important for the exposition of epitope and accessibility to antibodies [48].

**Table 1:** Sequence identity and similarity of the Cathepsin L-like protein (LbrM.08.0830) and B-cell linear epitopes predicted in the Cathepsin L-like sequence of *Leishmania braziliensis* and its orthologs.

Species	Cathepsin L-like		Peptide-1*	
	Identity	Similarity	Identity	Similarity
*Leishmania infantum*	65.99%	70.34%	40.00%	73.33%
*Trypanosoma cruzi*	47.60%	56.63%	53.30%	86.67%
*Canis familiaris*	28.12%	35.98%	0.00%	0.00%
*Homo sapiens*	28.34%	36.96%	0.00%	0.00%

*Peptide-1: QTSGSTTPGPTTTT.

**Figure 2:**
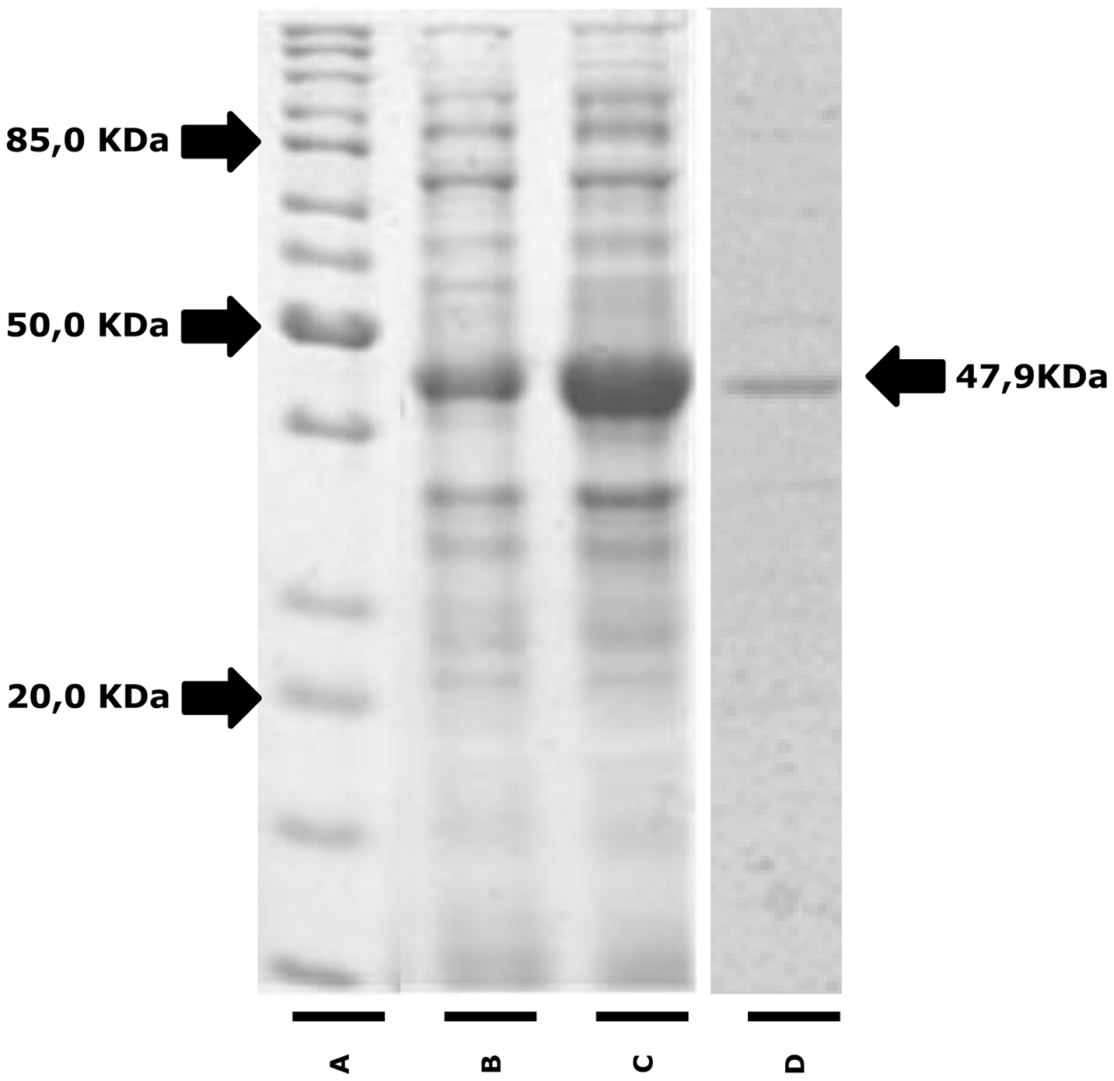
Expression and purification of recombinant Cathepsin L-like protein. Protein samples were separated by 12.5% SDS-PAGE gel electrophoresis. (**A**) Molecular weight standard, (**B**) lysate of culture before and (**C**) after induction with IPTG and (**D**) recombinant Cathepsin L-like protein (MW 47.6 KDa) purified by gel filtration.

The recombinant protein had a predicted molecular weight of 47.9 kDa, and it was successfully expressed and obtained at a high level of purity (Fig. 2). A peptide (QTSGSTTPGPTTTT) representing the predicted B-cell linear epitope was also chemically synthesized.

### Performance of *r*CatL and its linear epitope in the diagnosis of human leishmaniasis

**Figure 3:**
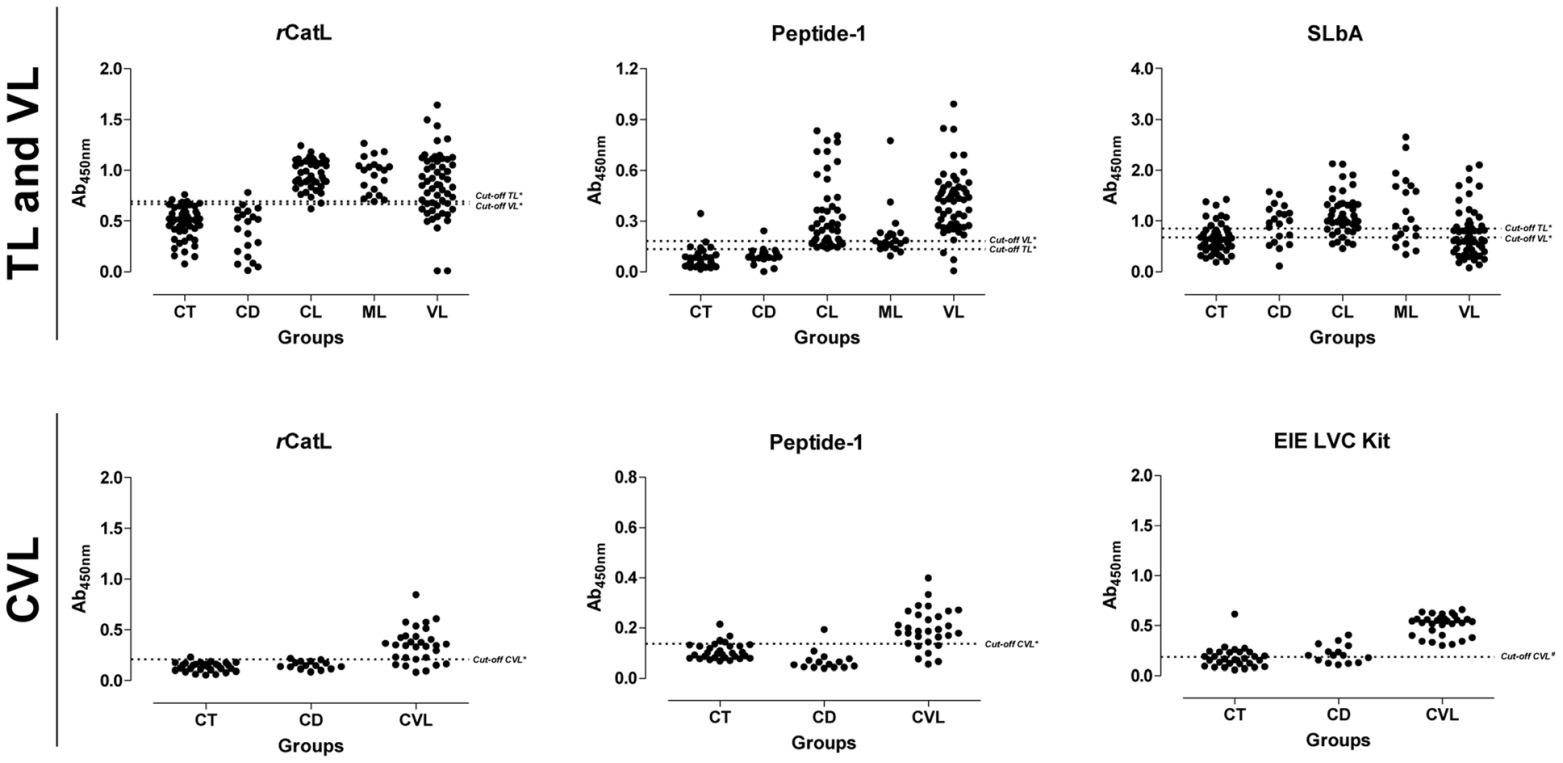
Comparison of the ELISA reactivity of *r*CatL, Peptide-1, SLbA and the EIE-LVC kit against sera from TL and VL patients and from *L. infantum*-infected dogs. **TL and VL**: ELISAs were performed on samples from different groups of individuals (CT, control group; CD, Chagas disease patients; CL, cutaneous leishmaniasis; ML, mucosal leishmaniasis; VL, visceral leishmaniasis). **CVL**: ELISAs were performed on samples from different groups of dogs (CT, control group; CD, *T. cruzi*-infected dogs; CVL, canine visceral leishmaniasis). ^*^Cut-off obtained by a ROC curve. ^#^Cut-off suggested by the manufacturer.

**Figure 4:**
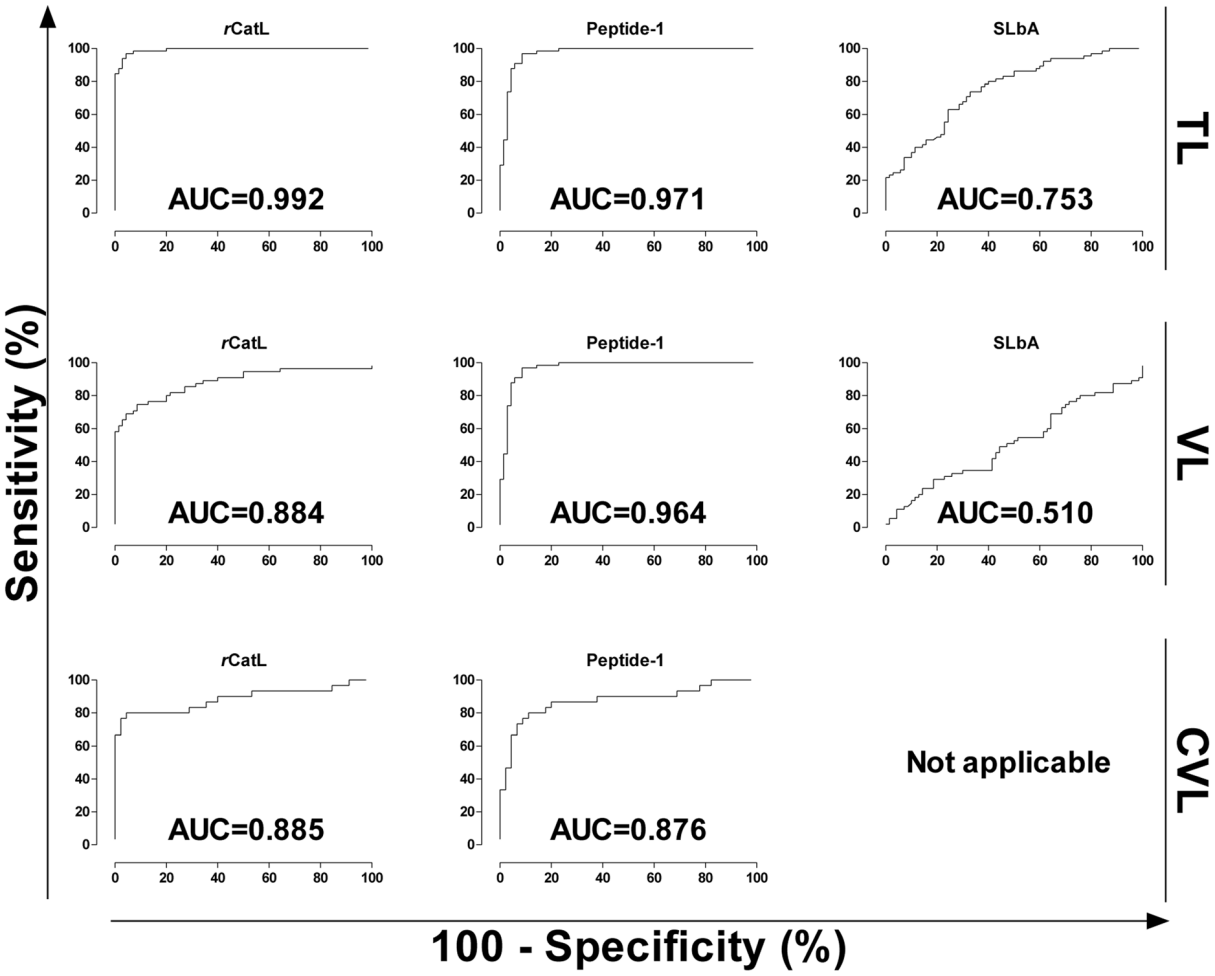
Comparison of ROC curves obtained from *r*CatL, Peptide-1 and SLbA. The ROC curves were used to determine the ELISA cut-off, sensitivity, specificity and AUC. In case of SLbA for CVL diagnosis (EIE-LVC Kit), ROC curve is not shown (not applicable) in this graph because the cut-off was determined according to the recommendations by the manufacturer (twice the average of the negative control included in kit).

*r*CatL, synthetic peptide and SLbA were evaluated for reactivity against sera from patients with tegumentary and visceral leishmaniasis (Fig. 3). Human tegumentary leishmaniasis included patients with cutaneous and mucosal clinical disease. Cross-reactivity with sera from chagasic patients was also evaluated. Samples from patients with mucosal disease had lower reactivity with the synthetic peptide than patients with cutaneous and visceral leishmaniasis, but this difference was not observed for the other antigens. The performance of each antigen (sensitivity, specificity, positive and negative predictive value and accuracy) is summarized in Table 2. Considering all metrics, the recombinant protein showed the best diagnostic value for tegumentary leishmaniasis (96.30% accuracy), followed by synthetic peptide, which also had a similarly high performance (above 90% for all parameters, including 94.07% accuracy). Analysis of area under curve (AUC) using ROC curves (Fig. 4) confirmed the better performance of *r*CatL (AUC = 0.992) and the synthetic peptide (AUC = 0.971) compared with SLbA (AUC = 0.753). Furthermore, these two antigens showed very good agreement with parasitological tests (Table 3), the gold standard for diagnosis of tegumentary leishmaniasis. Moreover, the soluble antigen had low sensitivity (70.77%), specificity (68.57%) and accuracy (69.63%) in diagnosing patients with cutaneous and mucosal disease.

**Table 2:** Diagnostic performance of *r*CatL, peptide-1, SLbA and the EIE-LVC kit.

Test	Disease	Cut-off	Parameters^a^						
			TSe (%)	CI 95%	TSp (%)	CI 95%	PPV (%)	NPV (%)	AC (%)
***r*** **CatL** *	TL	0.6916	96.92	89.32–99.63	95.71	87.98–99.11	95.45	97.10	96.30
**Peptide-1** *	TL	0.1340	96.92	89.32–99.63	91.43	82.27–96.79	91.30	96.97	94.07
**SLbA** *	TL	0.8530	70.77	58.17–81.40	68.57	56.37–79.15	67.65	71.64	69.63
***r*** **CatL** *	VL	0.6679	74.55	61.00–85.33	91.43	82.27–96.79	87.23	82.05	84.00
**Peptide-1** *	VL	0.1820	94.55	84.88–98.86	97.14	90.06–99.65	96.30	95.77	96.00
**SLbA** *	VL	0.6780	52.73	38.80–66.35	50.00	37.80–62.20	45.31	57.38	51.20
***r*** **CatL** *	CVL	0.2039	80.00	61.43–92.29	95.56	84.85–99.46	92.31	87.76	89.33
**Peptide-1** *	CVL	0.1363	80.00	61.43–92.29	88.89	75.95–96.29	82.76	86.96	85.33
**EIE-LVC Kit** #	CVL	0.1894	100.00	88.43–100.0	53.33	37.87–68.34	58.82	100.00	72.00

a Parameters was calculated using all samples presented in this work for TL (CT + CD + CL + ML. n = 135). VL (CT + CD + VL. n = 125) and CVL (CT + CD + CVL. n = 75).

*Cut-off obtained by ROC curve.

# *Cut off* obtained according to the manufacturer.

Abbreviations: Tse; total sensitivity; TSp: total specificity; CI: confidence interval; PPV: positive predictive value; NPV: negative predictive value; AC: accuracy.

**Table 3:** Diagnostic performance of *r*CatL, peptide-1, SLbA and the EIE-LVC kit using ROC curves. Data validation and agreement was confirmed using a kappa index.

Test	Disease	AUC	CI 95%	TP	TN	FP	FN	κ^a^	CI 95%	Agreement^b^
***r*** **CatL** *	TL	0.992	0.983–1.001	63	67	3	2	0.926	0.862–0.990	Very good
**Peptide-1** *	TL	0.971	0.943–0.998	63	64	6	2	0.882	0.802–0.961	Very good
**SLbA** *	TL	0.753	0.673–0.834	48	46	22	19	0.393	0.238–0.548	Fair
***r*** **CatL** *	VL	0.884	0.819–0.949	41	64	6	14	0.670	0.539–0.801	Good
**Peptide-1** *	VL	0.964	0.924–1.005	52	68	2	3	0.919	0.849–0.989	Very good
**SLbA** *	VL	0.510	0.405–0.614	29	35	35	26	0.027	−0.147–0.200	Poor
***r*** **CatL** *	CVL	0.885	0.794–0.976	24	43	2	6	0.773	0.625–0.920	Good
**Peptide-1** *	CVL	0.876	0.786–0.966	24	40	5	6	0.693	0.525–0.860	Good
**EIE-LVC Kit** #	CVL	NA	NA	30	24	21	0	0.478	0.316–0.639	Moderate

a The kappa index was calculated using all samples presented in this work for TL (CT + CD + CL + ML. n = 135), VL (CT + CD + VL. n = 125) and CVL (CT + CD + CVL. n = 75).

b Agreement was calculated using parasitological assays as the gold standard.

*Cut-off obtained by ROC curve.

# *Cut-off* suggested by the manufacturer.

Abbreviations: AUC: area under curve; CI: confidence interval; TP: true positive; TN: true negative; FP: false positive; FN: false negative; κ: kappa index; NA: not applicable.

Using samples from patients with visceral leishmaniasis, the synthetic peptide showed better performance values than *r*CatL and SLbA (Table 2); their accuracy values were 96.00, 84.00 and 51.20%, respectively. Of the metrics evaluated, sensitivity showed the highest difference between peptide (94.55%) and recombinant protein (74.55%). Only the synthetic peptide showed very good agreement with the parasitological test (Table 3), and the AUC for each antigen (0.884, *r*CatL; 0.964, peptide; 0.510, SLbA; Fig. 4) confirmed that the peptide could potentially be used for serodiagnosis of human visceral leishmaniasis.

### Performance of *r*CatL and its linear epitope in the diagnosis of canine leishmaniasis

The recombinant protein and synthetic peptide were also evaluated for the serodiagnosis of visceral canine leishmaniasis, and both were compared with the commercial EIE-LVC kit (Fig. 3). Although *r*CatL and the synthetic peptide had higher specificity than EIC-LVC, the commercial kit showed the highest sensitivity (Table 2). The accuracy value for *r*CatL (89.33%) was higher than synthetic peptide (85.33%) and EIE-LVC antigen (72.00%). The area under the curve for recombinant protein (0.885) was also higher than the peptide (0.876). Furthermore, these two antigens showed good agreement with parasitological tests, while the kit had only a moderate correlation (Table 3).

### Synthetic peptide representing the linear epitope is important for protein reactivity against sera from infected humans and dogs

**Figure 5:**
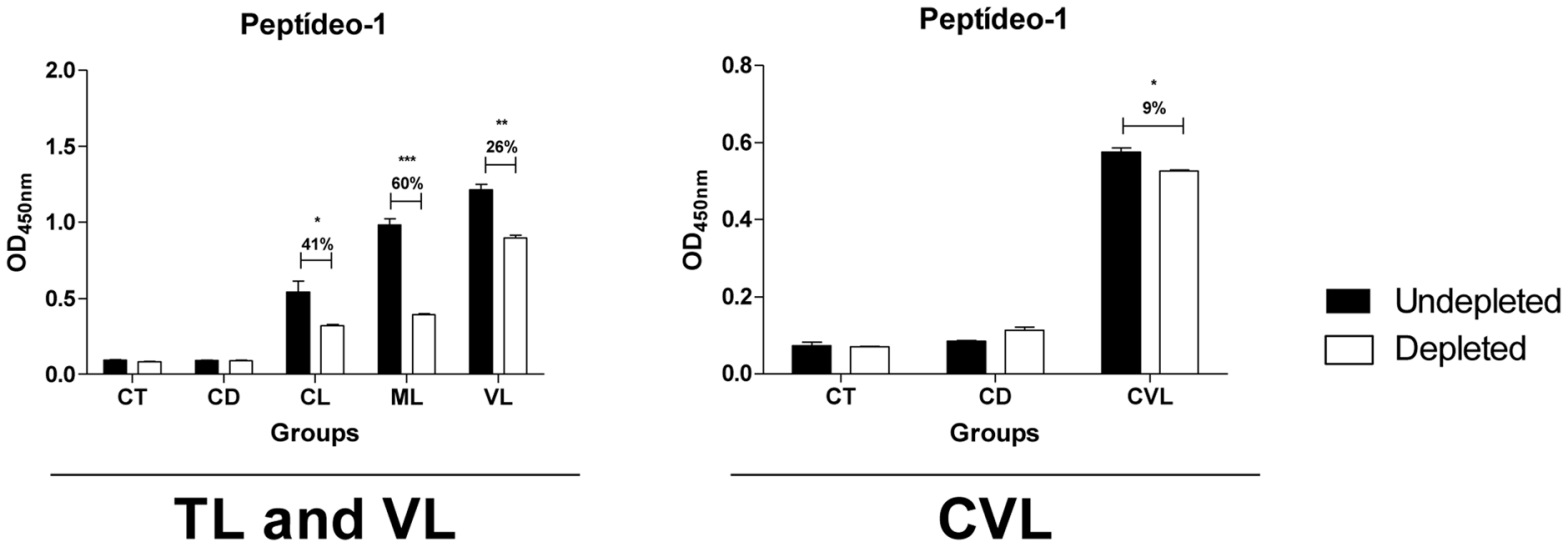
Immunodepletion assay showing specific IgG antibody recognition of the synthetic peptides with known reactivity to Cathepsin L-like. Pools of sera (n = 10) from the different groups were depleted with peptide-1 (CT, control group; CD, Chagas disease; CL, cutaneous leishmaniasis; ML, mucosal leishmaniasis; VL, visceral leishmaniasis; CVL, canine visceral leishmaniasis). The mean antibody OD values are shown on the *y*-axis, and the error bars indicate the standard deviation. Significant differences are indicated on the graphs (*p<0.05; **p<0.01; ***p<0.001).

Depletion ELISA was performed to confirm that the synthetic peptide represents a human and canine B-cell linear epitope in CatL. This technique is based on the reduction of serum reactivity via the depletion of peptide-specific antibodies; in this case, the sample is incubated with the synthetic peptide prior to ELISA with a known antigen [23], [24]. The depleted sample was tested for reactivity against the recombinant protein, and the reduction was proportional to antibody levels that bind to the similar peptide within the protein sequence. IgG reactivity against *r*CatL after antibody depletion was reduced in all *Leishmania*-infected human and dog groups (Fig. 5). Furthermore, the reduction in reactivity for both types of human tegumentary leishmaniasis (41%, *p*<0.05 for cutaneous and 60%, *p*<0.001 for mucosal) was higher than for human visceral disease (26%, *p*<0.01) and lower than the reduction in canine samples (9%, *p*<0.05). For the control groups and chagasic humans and dogs, no significant reduction was observed (Fig. 5), suggesting that this epitope is specific to *Leishmania* parasites.

## Discussion

Serological tests have significant advantages for leishmaniasis diagnosis. These tests allow the early detection of infection before lesion formation, and they are non-invasive, quantitative and easily automated, allowing the concurrent analysis of a large number of samples [17], [25], [49]. However, the specificity and sensitivity of current methods using crude antigens vary depending on antigen composition, parasite species and strain, production protocol and experimental conditions [11], [13]. For example, the soluble *L. braziliensis* antigen or the commercial EIE-LVC kit, which uses antigen prepared from *Leishmania major-like* promastigotes, both produce many false positive results. Thus, antigen selection is crucial for improving the specificity and sensitivity of the diagnostic technique. Recombinant protein antigens can be standardized and are safer than crude antigens because they do not require the maintenance and processing of live parasites. The recombinant K39 protein is the most promising protein; when used as a rapid test for visceral leishmaniasis, diagnosis reached 77–90% specificity and 87–93% sensitivity [50], [51]. The WHO Special Program for Research and Training in Tropical Disease (TDR) has evaluated five different immunodiagnostic tests using recombinant K39 or recombinant protein derived from the kinesin gene of *L. donovani* from East Africa, Brazil and India [52]. The sensitivities ranged from 36.8–100% and specificities from 90.8–100% with no test winner across all regions and conditions, thus demonstrating the importance of antigen identification for leishmaniasis serodiagnosis.

Because multiple *Leishmania* genomes have been sequenced, parasite protein sequences can be compared with those of their host and other pathogens to identify patterns associated to infection [53], including new targets for diagnosis. Proteins associated with infection and the intracellular survival of the parasite are attractive targets because they are generally secreted or expressed on the parasite surface [54]. Of these genes, CatL is a potential diagnostic target because it is expressed on the surface or secreted by intracellular parasites [29], [30], [34] and is involved in the infection of mammals. Importantly, the *L. braziliensis* CatL protein sequence has less than 50% identity with the orthologous proteins in humans, dog and *T. cruzi* but approximately 66% identity with the *L. infantum* protein. Furthermore, the predicted linear B-cell epitope in *L. braziliensis* CatL is absent from the human and dog proteins, reducing the potential of antigen cross-reactivity with non-infected hosts.

In context of the antigen-antibody or antigen-TCR (T-cell receptor) binding, the detection of specific amino acid residues that contribute to the specificity and strength of protein/peptide interactions is a problem of the utmost importance [25], [55]. In this sense, previous studies employing alanine scanning method indicates that there are several structural factors determined by physical-chemical properties of the amino acid sequence of the peptide that determine its affinity with epitope binding site [55]–[58]. Among these factors, the hydrophobic and electrostatic interactions they establish, as well as the flexibility of the molecules involved, are very significant [55]. Through similarity analysis, CatL B-cell epitope demonstrated to be more similar to the sequence in *T. cruzi* (86.67%) than in *L. infantum* (73.33%). However, experimental data obtained in this study for peptide-1 showed high sensitivity in the identification of infected-*L.infantum* individuals and high specificity in the discrimination of individuals infected with *T. cruzi*. These results together suggest that there are specific amino acids conserved only in *Leishmania* species, and the substitutions of some amino acids may imply a significant change in the affinity of antibody, as described in previous studies employing synthetic peptides [25]. In fact, only 1 of 20 chagasic patient sera and 1 of 15 *T. cruzi*-infected dog sera was reactive against the synthetic peptide above the cut-off, thus confirming the *Leishmania* specificity for this linear epitope. In agreement, the low cross-reactivity observed with the proteins of the host was important for obtaining high ability to discriminate infected individuals to controls.

The high conservation of proteins among the various *Leishmania* species opens the possibility for identification of an antigen able to simultaneously diagnose the various clinical forms of the disease would represent an interesting strategy for the technological development and large-scale production of tests for diagnosis [17]. In this sense, the present study was designed to identify antigens for multiple diagnosis of leishmaniasis.

Thus, due to low antibody titers observed in patients with TL in comparison to individuals with VL, we chose to select the protein present in the causative agent of TL (*L. braziliensis*) to ensure greater spectrum of diagnosis when employed in individuals infected by species that induce high production of antibodies, as *L. infantum* [14], [59]. Interestingly, we observed slight reduction in performance of the recombinant *L. braziliensis* CatL for diagnosis of the human visceral leishmaniasis when compared to respective synthetic peptide (accuracy value: 84.00% and 96.00%, respectively) while in the case of TL, performance data were similar. Based on these information, we speculate that the few amino acid substitutions that occurs in CatL sequence and its epitope in the native protein of different *Leishmania* species causing TL and VL, can trigger different exposure of the epitope due to conformational structure, and these characteristics associate the possibility of inducing selection of different B-cell clones producing of specific antibodies, can induce small variations in the recognition of the same epitope by individuals affected by different clinical forms of leishmaniasis, especially at the protein level.

Epitope prediction is a useful tool for screening and eliminating potential targets, which reduces research costs [27]. After epitope prediction, the experimental validation of peptide binding to antibodies specific to the original protein is important. For this purpose, depletion ELISA was performed in this study. After the depletion of antibodies that bound the peptide, both canine and human sera showed reactivity against the recombinant protein. This result confirmed the mapped epitope, as the reduced reactivity suggests that some antibodies in the sample reacted to the epitope in the protein and the synthetic peptide. However, the reactivity was not completely reduced, most likely because there are other non-predicted linear epitopes as well as conformational epitopes not accessed in this analysis.

Both recombinant protein and synthetic peptide were evaluated for their potential in *Leishmania* serodiagnosis. *r*CatL showed the best performance for the immunodiagnosis of human tegumentary and visceral canine disease, with specificities of 95.71 and 95.56% and sensitivities of 96.92% and 80%, respectively. The synthetic peptide was the best antigen for discriminating visceral leishmaniasis in human samples (94.55% sensitivity and 97.14% specificity). Notably, the re-calculation of performance metrics employing a balanced data (same number of individuals per group, randomly selected; S3–S4 Tables) did not return values significantly different from the unbalanced data (Tables 2 and 3), and the conclusions remain that *r*CatL is the best antigen for the immunodiagnosis of TL and CVL, and Peptide-1 for VL. An alternative method for the production of antigens for immunoassays is peptide synthesis. Peptides are relatively simple to synthesize, and they are less expensive and have fewer contaminants than recombinant proteins. Moreover, chemical synthesis protocols do not require the manipulation of living organisms. In general, synthetic peptides increase the specificity of immunoassays compared with crude antigens. The increased sensitivity and specificity of synthetic peptides is associated with specific immunogenic regions in parasite proteins and potential immunodominant proteins that are absent in the host or other organisms frequently associated with cross-reactivity. Both the recombinant protein and synthetic peptide showed higher specificity and sensitivity than crude preparations commonly used for other antigens [17], [52], and thus, they are valuable targets to compose an antigen panel that could significantly improve leishmaniasis diagnosis.

## Supporting Information

S1 Fig **Flow chart representing all human and canine sera samples used in the study. Human samples:** CT, control group, n = 50; CD, Chagas disease patients, n = 20; CL, cutaneous leishmaniasis, n = 45; ML, mucosal leishmaniasis, n = 20; VL, visceral leishmaniasis, n = 55. **Dog samples:** CT, control group, n = 30; CD, *T. cruzi*-infected dogs, n = 15; CVL, canine visceral leishmaniasis, n = 30.
(TIF)

S1 Table **Top 10 human proteins containing short sequences more similar to linear B-cell epitope of** ***L. braziliensis*** **CatL.**
(DOCX)

S2 Table **Top 10 dog proteins containing short sequences more similar to linear B-cell epitope of** ***L. braziliensis*** **CatL.**
(DOCX)

S3 Table **Diagnostic performance of rCatL, peptide-1, SLbA and the EIE-LVC kit using balanced data (n = 40 for TL, VL and n = 30 for CVL).**
(DOCX)

S4 Table **Diagnostic performance of rCatL, peptide-1, SLbA and the EIE-LVC kit using ROC curves and balanced data (n = 40 for TL, VL and n = 30 for CVL).** Data validation and agreement was confirmed using a kappa index.
(DOCX)
