# Supplementary material for: Improving Serodiagnosis of Human and Canine Leishmaniasis with Recombinant Leishmania braziliensis Cathepsin L-like Protein and a Synthetic Peptide Containing Its Linear B-cell Epitope
Source: PLoS Negl Trop Dis. 2015 Jan 8;9(1):e3426. doi: 10.1371/journal.pntd.0003426 (PMC4287388; doi:10.1371/journal.pntd.0003426)
Supplement: S2 Table — Top 10 dog proteins containing short sequences more similar to linear B-cell epitope of L. braziliensis CatL. (DOCX) [file pntd.0003426.s003.docx]

**Table S2.** Top 10 dog proteins containing short sequences more similar to linear B-cell epitope of *L. braziliensis* CatL.

| ***Canis familiares*** | | | |
| --- | --- | --- | --- |
| **Position** | **Gene ID** | **BLAST score** | **Similarity(%)** |
| 1 | gi\|402558\|emb\|CAA48914.1\| | 26.9 | 53.33 |
| 2 | gi\|402558\|emb\|CAA48914.1\| | 21.0 | 53.33 |
| 3 | gi\|402558\|emb\|CAA48914.1\| | 20.6 | 53.33 |
| 4 | gi\|402558\|emb\|CAA48914.1\| | 19.7 | 46.67 |
| 5 | gi\|402558\|emb\|CAA48914.1\| | 19.3 | 60.00 |
| 6 | gi\|402558\|emb\|CAA48914.1\| | 18.0 | 60.00 |
| 7 | gi\|402558\|emb\|CAA48914.1\| | 17.6 | 46.67 |
| 8 | gi\|402558\|emb\|CAA48914.1\| | 17.6 | 46.67 |
| 9 | gi\|402558\|emb\|CAA48914.1\| | 16.3 | 60.00 |
| 10 | gi\|402558\|emb\|CAA48914.1\| | 15.9 | 40.00 |
