# Supplementary material for: Improving Serodiagnosis of Human and Canine Leishmaniasis with Recombinant Leishmania braziliensis Cathepsin L-like Protein and a Synthetic Peptide Containing Its Linear B-cell Epitope
Source: PLoS Negl Trop Dis. 2015 Jan 8;9(1):e3426. doi: 10.1371/journal.pntd.0003426 (PMC4287388; doi:10.1371/journal.pntd.0003426)
Supplement: S3 Table — Diagnostic performance of rCatL, peptide-1, SLbA and the EIE-LVC kit using balanced data (n = 40 for TL, VL and n = 30 for CVL). (DOCX) [file pntd.0003426.s004.docx]

**Table S3.** Diagnostic performance of *r*CatL, peptide-1, SLbA and the EIE-LVC kit using balanced data (n=40 for TL, VL and n=30 for CVL).

| **Test** | **Disease** | **Cut-off** | **Parameters*^a^*** | | | | | | |
| --- | --- | --- | --- | --- | --- | --- | --- | --- | --- |
|  |  |  | **TSe (%)** | **CI 95%** | **TSp (%)** | **CI 95%** | **PPV (%)** | **NPV (%)** | **AC (%)** |
| ***r*CatL*** | TL | 0.6836 | 100.00 | 91.19-100.00 | 95.00 | 83.08-99.39 | 95.23 | 100.00 | 97.50 |
| **Peptide-1*** | TL | 0.1340 | 95.00 | 83.08-99.39 | 92.50 | 79.61-98.43 | 92.68 | 94.87 | 93.75 |
| **SLbA*** | TL | 1.1710 | 45.00 | 29.26-61.51 | 87.50 | 73.20-95.81 | 78.26 | 61.40 | 66.25 |
| ***r*CatL*** | VL | 0.6679 | 75.00 | 58.80-87.31 | 92.50 | 79.61-98.43 | 90.90 | 78.72 | 83.75 |
| **Peptide-1*** | VL | 0.1820 | 95.00 | 83.08-99.39 | 97.50 | 86.84-99.94 | 97.43 | 95.12 | 96.25 |
| **SLbA*** | VL | 0.8231 | 65.00 | 48.32-79.37 | 45.00 | 29.26-65.51 | 68.42 | 56.25 | 55.00 |
| ***r*CatL*** | CVL | 0.2039 | 80.00 | 61.43-92.29 | 96.67 | 82.78-99.92 | 96.00 | 82.86 | 88.33 |
| **Peptide-1*** | CVL | 0.1272 | 80.00 | 61.43-92.29 | 90.00 | 73.47-97.89 | 88.89 | 81.81 | 85.00 |
| **EIE-LVC Kit^#^** | CVL | 0.1894 | 100.00 | 88.43-100.00 | 40.00 | 22.66-59.40 | 62.50 | 100.00 | 70.00 |
| *^a^*Parameters was calculated using all samples presented in this work for TL (CT + CD = 40; CL + ML = 40), VL (CT +CD = 40; VL = 40) and CVL (CT + CD = 30; CVL = 30). | | | | | | | | | |
|  |  |  |  |  |  |  |  |  |  |
| ** Cut-off* obtained by ROC curve. | | | | | | | | | |
| *^#^ Cut off* obtained according to the manufacturer. | | | | | | | | | |
| Abbreviations: Tse; total sensitivity; TSp: total specificity; CI: confidence interval; PPV: positive predictive value; NPV: negative predictive value; AC: accuracy. | | | | | | | | | |
|  |  |  |  |  |  |  |  |  |  |
