# Supplementary material for: Improving Serodiagnosis of Human and Canine Leishmaniasis with Recombinant Leishmania braziliensis Cathepsin L-like Protein and a Synthetic Peptide Containing Its Linear B-cell Epitope
Source: PLoS Negl Trop Dis. 2015 Jan 8;9(1):e3426. doi: 10.1371/journal.pntd.0003426 (PMC4287388; doi:10.1371/journal.pntd.0003426)
Supplement: S4 Table — Diagnostic performance of rCatL, peptide-1, SLbA and the EIE-LVC kit using ROC curves and balanced data (n = 40 for TL, VL and n = 30 for CVL). Data validation and agreement was confirmed using a kappa index. (DOCX) [file pntd.0003426.s005.docx]

**Table S4.** Diagnostic performance of *r*CatL, peptide-1, SLbA and the EIE-LVC kit using ROC curves and balanced data (n=40 for TL, VL and n=30 for CVL). Data validation and agreement was confirmed using a kappa index.

| **Test** | **Disease** | **AUC** | | **CI 95%** | **TP** | | **TN** | | **FP** | | **FN** | | **κ*^a^*** | | **CI 95%** | | **Agreement*^b^*** | |  |
| --- | --- | --- | --- | --- | --- | --- | --- | --- | --- | --- | --- | --- | --- | --- | --- | --- | --- | --- | --- |
| ***r*CatL*** | TL | 0.9931 | 0.9818-1.004 | | | 40 | | 38 | | 2 | | 0 | | 0.950 | | 0.882-1.000 | | Very good | |
| **Peptide-1*** | TL | 0.9719 | 0.9415-1.002 | | | 38 | | 37 | | 3 | | 2 | | 0.875 | | 0.769-0.981 | | Very good | |
| **SLbA*** | TL | 0.6950 | 0.5800-0.8100 | | | 18 | | 35 | | 5 | | 22 | | 0.325 | | 0.137-0.513 | | Fair | |
| ***r*CatL*** | VL | 0.8738 | 0.7940-0.9535 | | | 30 | | 37 | | 3 | | 10 | | 0.675 | | 0.516-0.834 | | Good | |
| **Peptide-1*** | VL | 0.9681 | 0.9197-1.017 | | | 38 | | 39 | | 1 | | 2 | | 0.925 | | 0.842-1.000 | | Very good | |
| **SLbA*** | VL | 0.5156 | 0.3875-0.6438 | | | 26 | | 18 | | 12 | | 14 | | 0.248 | | 0.020-0.475 | | Fair | |
| ***r*CatL*** | CVL | 0.8889 | 0.7270-1.033 | | | 24 | | 29 | | 1 | | 6 | | 0.767 | | 0.606-0.927 | | Good | |
| **Peptide-1*** | CVL | 0.8800 | 0.7908-0.9692 | | | 24 | | 27 | | 3 | | 6 | | 0.700 | | 0.520-0.880 | | Good | |
| **EIE-LVC Kit^#^** | CVL | NA | NA | | | 30 | | 12 | | 18 | | 0 | | 0.400 | | 0.214-0.586 | | Fair | |
| *^a^*The kappa index was calculated using all samples presented in this work for TL (CT + CD = 40; CL + ML = 40), VL (CT +CD = 40; VL = 40) and CVL (CT + CD = 30; CVL = 30). | | | | | | | | | | | | | | | | | | |  |
|  |  |  |  |  |  |  |  |  |  |  |  |  |  |  |  |  |  |  |  |
| *^b^*Agreement was calculated using parasitological assays as the gold standard. | | | | | | | | | | | | | | | | | | |  |
| ** Cut-off* obtained by ROC curve. | | | | | | | | | | | | | | | | | | |  |
| *^#^ Cut-off* suggested by the manufacturer. | | | | | | | | | | | | | | | | | | |  |
| Abbreviations: AUC: area under curve; CI: confidence interval; TP: true positive; TN: true negative; FP: false positive; FN: false negative; κ: kappa index; NA: not applicable. | | | | | | | | | | | | | | | | | | |  |
